# Effectiveness of Mycophenolate Mofetil in the Treatment of Pediatric Anti-NMDAR Encephalitis: A Retrospective Analysis of 6 Cases

**DOI:** 10.3389/fneur.2020.584446

**Published:** 2020-11-09

**Authors:** Xiao-sheng Hao, Jiang-tao Wang, Chen Chen, Yun-peng Hao, Jian-min Liang, Song-yan Liu

**Affiliations:** ^1^The China-Japan Union Hospital, Changchun, China; ^2^The First Hospital of Jilin University, Changchun, China

**Keywords:** anti-N-methyl-D-aspartate receptor encephalitis, mycophenolate mofetil, pediatrics, efficacy, safety

## Abstract

**Objective:** To explore the effectiveness and safety of mycophenolate mofetil (MMF) as a second-line medication in the treatment of anti-N-methyl-D-aspartate receptor (NMDAR) encephalitis, the most common and severe autoimmune encephalitis.

**Methods:** The clinical data of six children with anti-NMDAR encephalitis admitted to the First Hospital of Jilin University were retrospectively analyzed, and the effectiveness and safety of MMF were evaluated.

**Results:** Six children with anti-NMDAR encephalitis were treated with MMF in the 2nd or 3rd treatment disease event (3 cases vs. 3 cases). MMF initiation was mean 19.2 months (range 6–39 months) after disease onset at a mean dose of 25.6 mg/kg (range 19.6–28.4 mg/kg) for 14 months (range 6–26 months). Only two patients had transient mild diarrhea within 2 weeks of MMF application. During follow-up, one patient relapsed whilst on MMF, one patient discontinued MMF, and 4 cases were still on MMF.

**Conclusion:** The use of MMF in anti-NMDAR encephalitis may be effective and safe. MMF can be used as one of the relapse prevention options in patients who already have relapsed or possibly even after the first event. Delayed use may be the main reason for MMF failure.

## Introduction

Anti-N-methyl-D-aspartate receptor (NMDAR) encephalitis is the most common autoimmune encephalitis characterized by complex neuropsychiatric syndromes and the presence of anti-GluN1 (NMDAR subunit NR1) antibodies in the cerebrospinal fluid (CSF) and serum (1–3). Since the anti-NMDAR autoantibody is the main cause of anti-NMDAR encephalitis, therapeutic approaches that lead to the removal of these antibodies can be utilized as the treatment of this severe encephalitis (4). The first-line treatments including intravenous methylprednisolone (suppressing the immune system), intravenous immunoglobulin (IVIG), and plasma exchange (physically removing the autoantibodies) can be effective in 53–80% of patients. The second-line treatments can be initiated when the first-line treatments are not effective within 10–14 days (5, 6). Second-line medications include rituximab (RTX), cyclophosphamide (CYC), azathioprine (AZA), and mycophenolate mofetil (MMF) (7–9). Among them, RTX is the most frequently used second-line agent. The use of MMF and AZA is more prevalent in Europe than in the USA (15 vs. 5%) (8).

MMF and mycophenolate sodium are both converted to mycophenolic acid, which can be utilized to treat immune-related diseases such as systemic lupus erythematosus, autoimmune vasculitis, inflammatory bowel disease, systemic sclerosis, inflammatory myopathy, and kidney or skin disorders (10). MMF can function as a potent inhibitor of lymphocyte proliferation through the reversible inhibition of adenosine monophosphate dehydrogenase and reduce antibody production. Due to the excellent immunosuppressive effects and economical price, MMF has been increasingly used in the treatment of neurological diseases such as neuromyelitis optica spectrum disorders, myelin oligodendrocyte glycoprotein (MOG)-associated disease, and multiple sclerosis (11). However, clinical studies on the treatment of anti-NMDAR encephalitis with MMF are still limited. By using a retrospective analysis of clinical features and therapies of six children suffering anti-NMDAR encephalitis, we investigated the effectiveness and side effects of MMF in the management of this severe autoimmune disease.

## Patients and Methods

In our study, anti-NMDAR encephalitis was mainly diagnosed based on clinical symptoms (including abnormal behavior/cognition, memory deficit, speech disorder, loss of consciousness, movement disorders, seizures, and autonomic dysfunction) and positive anti-GluN1 (NMDAR subunit NR1) antibodies in the cerebrospinal fluid or/and blood serum (3). In total, 6 children with anti-NMDAR encephalitis treated with MMF at the Department of Pediatric Neurology of the First Hospital of Jilin University from January 2015 to May 2020 were included in this study. Patients' personal information (gender and age), clinical symptoms, first-disease event auxiliary examinations [e.g., CSF biochemistry, anti-NMDAR antibody, oligoclonal bands (OCB), electroencephalogram (EEG), head magnetic resonance imaging (MRI)], treatments, and follow-up were collected, recorded, and retrospectively evaluated. Since MMF belongs to off-label medication, informed consents from guardians/parents have been obtained before using this medication. In addition, the study protocol was approved by the Ethics Committee of the First Hospital of Jilin University. Written informed consent was obtained from the minor(s)' legal guardian for the publication of any potentially identifiable images or data included in this article.

## Results

### Clinical Manifestations

In this study, there were 6 children (Table 1) including 5 girls and 1 boy and the mean age was 7.1 years (range 3–12 years). Clinical symptoms during the first disease event of disease included seizures (5 cases), abnormal (psychiatric) behavior (5 cases), movement disorder (4 cases), speech dysfunction (4 cases), fever (3 cases), headache (2 cases), and sleep disorder (2 cases). The initial symptoms were seizures (3 cases), fever (2 cases), and dyskinesia (1 case). All the 6 cases had the second relapses and clinical symptoms included seizures (4 cases), abnormal (psychiatric) behavior (3 cases), speech disorder (2 cases), headache (2 cases), and fever (1 case). Third relapses occurred in 3 patients, and the clinical symptoms included convulsion (1 case), abnormal (psychiatric) behavior (1 case), and fever (1 case).

**Table 1:** Clinical data of 6 cases with anti-NMDAR encephalitis treated with MMF.

		**Case 1**	**Case 2**	**Case 3**	**Case 4**	**Case 5**	**Case 6**
**Gender**		**Female**	**Female**	**Female**	**Male**	**Female**	**Female**
	Age at onset (years)	8	7	3	12	5	8
First disease event	Initial symptoms	Fever	Involuntary movements of the right hand	Seizures	Fever	Seizures	Seizures
	Other symptoms	Headache	Seizures, dysphoria, abnormal postures, verbal reduction	Dysphoria, involuntary movement, verbal reduction, sleep disorder	Seizures, Dysphoria	Movement disorder, dysphoria, verbal reduction	Dysphoria, movement disorder, verbal reduction, sleep disorder, fever, Headache
	mRS score	1	4	4	3	4	4
	Second- line therapy	–	RTX	–	–	–	–
WBC in CSF (× 10^6^/L)		120	32	10	19	126	44
Mononuclear cells		0.82	0.99	0.99	0.95	0.99	0.89
polykaryocyte		0.18	0.01	0.01	0.05	0.01	0.11
Anti-NMDAR antibody in serum		+	–	–	+	–	+
Anti-NMDAR antibody in CSF		+	+	+	+	+	+
Head MRI		Abnormal	Normal	Normal	Normal	Normal	Normal
EEG		Abnormal	Abnormal	Abnormal	Abnormal	Abnormal	Abnormal
Length of hospital stay (days)		22	23	15	27	22	33
Second disease event	Interval between first episode	17 mo	8 mo	16 mo	6 mo	10 mo	3 mo
	Clinical manifestations	Fever, Headache	Seizures	Seizures, dysphoria, mutism	Dysphoria	Seizures, dysphoria, mutism	Seizures, Headache
	mRS	1	3	4	3	4	3
	Second- line therapy	–	MMF	MMF	MMF	RTX	RTX
Third disease event	Interval between first episode	39 mo	–	–	–	16 mo	30 mo
	Clinical manifestations	Fever	–	–	–	Dysphoria	Seizures
	mRS	1	–	–	–	4	3
	Second- line therapy	MMF	–	–	–	MMF	MMF
MMF dosage (mg/kg/d)		19.6	27.8	28.4	25.7	25.0	26.9
MMF concentration^*^ (mg/L)		3.42	3.01	3.37	2.79	3.16	3.48
Trimethoprim/sulfamethoxazole (Calculated with sulfamethoxazole) (mg/kg/d)		31.4	39.3	36.4	41.1	40.0	43.0
MMF treatment time		12 mo	6 mo	14 mo	8 mo	18 mo	26 mo
MMF treatment status		Discontinued	In use	In use	In use	In use	Stopped
Adverse effects		None	None	None	Diarrhea	None	Diarrhea
Clinical manifestations in last follow-up		Fever	–	Verbal reduction, intermittent dysphoria	–	Verbal reduction	–
mRS		1	0	2	0	3	0

*mRS, modified Rankin Scale; RTX, rituximab; MMF, mycophenolate mofetil; CSF, cerebrospinal fluid; MRI, magnetic resonance imaging; EEG, electroencephalogram.*

* *MMF concentration measured during the 1st month after MMF initiation (normal range: 1.0–3.5 mg/L)*.

### Auxiliary Examinations

In all 6 cases, the CSF white blood cell (WBC) count was increased with a mean value of 59 × 10^6^/L (range, 10–126 × 10^6^/L) and differential showed mononuclear cell predominance. CSF total protein, glucose, and chloride were within the normal range for all 6 cases. Anti-NMDAR antibodies were positive in all 6 cases' CSF (Cell-based assay), and only in 3 cases' serum. Case 2 and Case 3's CSF OCBs were positive, while their serum OCBs were negative. For all 6 cases anti-MOG, anti-aquaporin-4, and paraneoplastic antibodies in both serum and CSF were negative. Thoracic and abdominal CT scans were normal in all 6 cases. Head MRI scans were normal in 5 cases and abnormal in 1 case (having patchy abnormal signals in the right pons arm and left frontal and parietal lobes). All EEGs were abnormal and main manifestations included slowed background activities (6 cases), focal slow waves (5 cases), focal epileptic waves (3 cases), a left temporal region initiation (Case 4). There was no δ brush (transient patterns characterized by a slow delta wave with superimposed fast activity) in all EEGs.

### Treatment and Prognosis

All patients were treated with corticosteroid (i.v. methylprednisone 15 mg/kg/d, maximum dose of 1,000 mg, once daily for 3 days, and then oral prednisone 1.5–2 g/kg/d for 4 days; cycle weekly for 3 weeks) and IVIG (2 g/kg divided over 3–5 days) during each acute phase. After that, oral prednisone (1.5–2 g/kg/d, with gradual tapering) was administered for 3–6 months. One patient (Case 2) was given RTX during the first disease event, while two patients (Case 5 and Case 6) received RTX during the second disease event. MMF (dispersible tablets) was given to 3 patients during the second disease event and 3 patients during the third disease event. The mean duration of MMF initiation was 19.2 months (range, 6–39 months) after disease onset at a mean dose of 25.6 mg/kg (range 19.6–28.4 mg/kg) for 14 months (range, 6–26 months). Oral trimethoprim/sulfamethoxazole (calculated with sulfamethoxazole, 31.4–43.0 mg/kg/d for 3 days each week) was administered during the therapeutic periods of MMF and prednisone. Case 1 discontinued MMF due to relapse 12 months after MMF initiation with adequate doses, and the patient presented with fever and abnormalities on brain MRI 12 months after MMF initiation. Case 6 discontinued MMF after 26 months of asymptomatic application and felt no discomfort for 7 months. The remaining 4 cases were still receiving MMF and no relapse occurred. Cases 2–6 had a declining modified Rankin Scale (mRS). However, during follow-up, Case 3 continued to have verbal reduction and intermittent dysphoria, and Case 5 only had verbal reduction. The other patients had no adverse effects, except transient mild diarrhea during 2 weeks of MMF application in Case 4 and Case 6.

## Discussion

Approximately 66–80% of patients with anti-NMDAR encephalitis recover or have mild sequelae. However, all other patients may suffer from severe deficits such as neuropsychiatric or cognitive deficits, or death (5). The management of anti-NMDAR encephalitis remains a challenge. Methylprednisolone is a widely used first-line drug. However, the optimal dosage, time and frequency have not been clarified. In China, some physicians recommend a dose of 20–30 mg/kg/d, while others recommend 15–30 mg/kg/d, with the maximum dose of ≤ 1,000 mg. In our clinical practice, we usually choose 15–20 mg/kg/d, although systematic clinical validation has not been conducted. In addition to first-line drugs, many drugs including second-line drugs, are under continuous clinical research. We have found that MMF is effective and safe in the treatment of six children with anti-NMDAR encephalitis.

Despite the severity of anti-NMDAR encephalitis, early diagnosis and combinatorial therapy can improve the patients' outcomes. If first-line treatment is ineffective or the diagnosis is delayed, second-line treatment should be initiated as early as possible. RTX and CYC are the most frequently recommended second-line drugs that can be taken intravenously. MMF can be used when: (a) it is available as a second-line drug and second-line therapy is ineffective; (b) first-line therapy is effective, but IVIG or steroids cannot be tapered off (7); (c) there are antibody-negative situations, or unknown immunization status (7).

The starting time of MMF application is variable between patients of autoimmune encephalitis. Due to MMF's slow onset of action, it may take at least 2 months for this treatment to work. Therefore, many studies suggested that the duration of MMF treatment should be at least 6 months to more than 1 year (12, 13). Nosadini et al. (14, 15) reported that MMF was started at a mean time of 17.9–21.4 months after onset, and the mean duration of MMF treatment was 14.2–23.2 months. In our study, based on responsiveness to first-line therapy, 3 patients received RTX. However, all these 3 patients experienced relapses and were then treated with MMF. The start time of MMF treatment was mean 19.2 months (range 6–39 months) after onset, and the mean duration of treatment was 14 months (range 6–26 months).

27.3–85% of anti-NMDAR encephalitis can cause functional impairments, with a recurrence rate of 12–29% and a median interval time of 2 years (5). Before MMF treatment, the mean number of recurrences was 2.1 times (range 1–11 times). After MMF treatment, there was no recurrence (15). In a single-center longitudinal study of 220 patients (including 69 patients (31.4%) younger than 18 years old) with anti-NMDAR encephalitis (16), a higher proportion of patients (109 cases, 49.5%) received MMF treatment. Relapses were observed in 80 patients, with 21 patients having multiple relapses (2–4 times) and the median duration from onset to the first relapse was 7 months (interquartile range [IQR], 5–10 months), which indicated that long-term MMF might not prevent further recurrences.

All children in our study experienced 1–2 relapses before MMF treatment. The first relapse interval after the initial episode was mean 10 months (range 3–17 months), and the second relapse was mean 28.3 months (6–27 months). At the end of the present study, one patient had relapsed, another patient discontinued for 7 months without relapse, and the remaining four cases are still in use. Recent studies showed a few features of the patients having relapses despite the MMF treatment, compared with the MMF-treated patients without relapses (14). The first was that these patients tended to be younger (4 years 2 months vs. 7 years 7 months). The second was that these patients had more relapses (2.5 times vs. 1 time) before MMF treatment. The third was that their MMF treatment was later (>6 months after onset). The last was that MMF doses were lower than those of patients without relapses.

Patients tended to have a lower recurrence rate if they received the first and/or second-line treatment within 30 days of onset or MMF treatment earlier (especially within 3 months after onset) (15). Another study showed that delayed MMF treatment might be the main reason for its failure (17). Since MMF was not widely regarded as a second-line drug in the treatment of anti-NMDAR encephalitis, it was often administered when other treatments failed, or multiple relapses occurred, which might reduce the effectiveness of MMF treatment. In our study the relapses of Case 1 may be related to the late initiation of MMF (39 months from onset).

In the MMF instructions, the recommended dose for children is 600 mg/m^2^ orally twice a day (up to a maximum of 1 g, twice daily). However, many drugs in children are dosed according to bodyweight (mg/kg). The dose of MMF can be up to 35–50 mg/kg/d (14), with a maximum dose of 2–2.5 g/d (11). In our study, MMF (20–30 mg/kg/day) was used in our hospital and our dose was equal to 19.6–28.4 mg/kg. Since the dosage in the manual was determined mainly on the basis of pharmacokinetics and safety data of children after kidney transplantation, and then converted to mg/kg, it may be generally lower than needed in other diseases and conditions. Therefore, high-quality cohort studies are still needed to find out whether this recommended dosage is suitable and effective for neurological autoimmune diseases such as autoimmune encephalitis (18).

Common adverse events of MMF include diarrhea, myelosuppression, infection, and tumor formation (17). Firstly, diarrhea is the most common adverse event. In our study, we have observed mild diarrhea in 2 cases. However, it can be gradually tolerated, and there is no need to stop MMF in most conditions. Secondly, myelosuppression can be monitored early by routine blood tests, which should be performed 1–2 weeks after the onset of treatment, and every 6–8 weeks thereafter. Thirdly, infection can occur in some patients. Therefore, we usually performed screening of hepatitis B virus, hepatitis C virus, and tuberculosis at an early stage to rule out potential viral infections or tuberculosis. Lastly, long-term MMF treatment may lead to tumor formation since the immune system is suppressed.

The prophylactic treatment for pneumocystis carinii pneumonia (PCP) remains controversial. Although PCP treated with MMF and steroids has been reported in previous studies (19), the prophylactic treatment of PCP is not routine in patients after kidney transplantation (20). The indications for prophylactic treatment of PCP include: (a) the usage of two or more immunosuppressive agents; (b) the presence of underlying rheumatic diseases (such as systemic lupus erythematosus, idiopathic inflammatory myopathy) or opportunistic infections (such as cytomegalovirus); and (c) a daily dosage of MMF exceeding 3 g/d. For the prophylactic treatment of PCP, a combination of TMP and SMX is recommended (11). In our practice, oral TMP-SMX is administrated during the usage of MMF and prednisone. TMP-SMX is discontinued when the steroids are withdrawn (3–6 months).

It is controversial whether routine blood concentration monitoring of MMF is required. Routine monitoring of MMF may not be necessary for conventional doses, but is usually required for concomitant use with other drugs such as cyclosporine and hormones (19, 21). In all six patients in this study, the blood concentration of MMF was measured within 1 month after MMF initiation. Thereafter, considering the safety of MMF, no continuous monitoring was performed.

Early diagnosis and treatment can improve the prognosis of patients with anti-NMDAR encephalitis (22, 23). However, recurrence remains a problem for both patients and physicians. The underlying causes are unknown, and the use of immunosuppressive agents is still being explored. The major limitations of our study were the small sample size and single-center, retrospective nature of the cohort. Despite these constraints, the present case series indicates that MMF may be effective and safe for the treatment of pediatric anti-NMDAR encephalitis. Further clinical studies should be performed to optimize the MMF treatment in order to achieve better outcomes for this severe autoimmune encephalitis.

## Data Availability Statement

The raw data supporting the conclusions of this article will be made available by the authors, without undue reservation.

## Ethics Statement

The studies involving human participants were reviewed and approved by this study was approved by the ethics committee of The First Hospital of Jilin University. All procedures performed in studies involving human participants were in accordance with the ethical standards of the institutional and/or national research committee and with the 1964 Helsinki declaration and its later amendments or comparable ethical standards. The parents of patients/participants provided their written informed consent to participate in this study.

## Author Contributions

X-sH, J-mL, and S-yL designed/performed most of the investigation, data analysis, and wrote the manuscript. J-tW and CC contributed to interpretation of the data and analyses. Y-pH collected the data. All of the authors have read and approved the manuscript.

## Conflict of Interest

The authors declare that the research was conducted in the absence of any commercial or financial relationships that could be construed as a potential conflict of interest.

## Abbreviations

NMDAR: N-methyl-D-aspartate receptor
MOG: myelin oligodendrocyte glycoprotein
OCB: oligoclonal bands
CSF: cerebrospinal fluid
WBC: white blood cell
EEG: electroencephalogram
MRI: magnetic resonance imaging
mRS: modified Rankin Scale
MMF: mycophenolate mofetil
RTX: rituximab
CYC: cyclophosphamide
AZA: azathioprine
IVIG: intravenous immunoglobulin.
