# Corrigendum: Effectiveness of mycophenolate mofetil in the treatment of pediatric anti-NMDAR encephalitis: A retrospective analysis of 6 cases

**DOI:** 10.3389/fneur.2023.1178132

**Published:** 2023-03-14

**Authors:** Xiao-sheng Hao, Jiang-tao Wang, Chen Chen, Yun-peng Hao, Jian-min Liang, Song-yan Liu

**Affiliations:** ^1^The China-Japan Union Hospital, Changchun, China; ^2^The First Hospital of Jilin University, Changchun, China

**Keywords:** anti-N-methyl-D-aspartate receptor encephalitis, mycophenolate mofetil, pediatrics, efficacy, safety

In the published article, there was an error regarding the affiliations. Instead of “1 The First Hospital of Jilin University, Changchun, China, 2 The China-Japan Union Hospital, Changchun, China”, it should be “1 The China-Japan Union Hospital, Changchun, China, 2 The First Hospital of Jilin University, Changchun, China”.

There was also an error regarding the affiliations for Xiao-sheng Hao. As well as having the affiliation “The First Hospital of Jilin University, Changchun, China”, they should also have the affiliation, “The China-Japan Union Hospital, Changchun, China”.

The authors apologize for this error and state that this does not change the scientific conclusions of the article in any way. The original article has been updated.

